# Microsatellite instability and immune checkpoint inhibitors: toward precision medicine against gastrointestinal and hepatobiliary cancers

**DOI:** 10.1007/s00535-019-01620-7

**Published:** 2019-09-07

**Authors:** Yuji Eso, Takahiro Shimizu, Haruhiko Takeda, Atsushi Takai, Hiroyuki Marusawa

**Affiliations:** 1 Department of Gastroenterology and Hepatology, Graduate School of Medicine, Kyoto University, 54 Shogoin-Kawaharacho, Sakyo-ku, Kyoto, 6068507 Japan; 2 Department of Gastroenterology and Hepatology, Osaka Red Cross Hospital, 5-30 Fudegasaki-cho, Tennoji-ku, Osaka, 5438555 Japan

**Keywords:** Gastric cancer, Hepatocellular carcinoma, Immune checkpoint inhibitor, Microsatellite instability, Pancreatic cancer

## Abstract

Recent innovations in the next-generation sequencing technologies have unveiled that the accumulation of genetic alterations results in the transformation of normal cells into cancer cells. Accurate and timely repair of DNA is, therefore, essential for maintaining genetic stability. Among various DNA repair pathways, the mismatch repair (MMR) pathway plays a pivotal role. MMR deficiency leads to a molecular feature of microsatellite instability (MSI) and predisposes to cancer. Recent studies revealed that MSI-high (MSI-H) or mismatch repair-deficient (dMMR) tumors, regardless of their primary site, have a promising response to immune checkpoint inhibitors (ICIs), leading to the approval of the anti-programmed cell death protein 1 monoclonal antibody pembrolizumab for the treatment of advanced or recurrent MSI-H/dMMR solid tumors that continue to progress after conventional chemotherapies. This new indication marks a paradigm shift in the therapeutic strategy of cancers; however, when considering the optimum indication for ICIs and their safe and effective usage, it is important for clinicians to understand the genetic and immunologic features of each tumor. In this review, we describe the molecular basis of the MMR pathway, diagnostics of MSI status, and the clinical importance of MSI status and the tumor mutation burden in developing therapeutic strategies against gastrointestinal and hepatobiliary malignancies.

## Introduction

In recent years, immune checkpoint inhibitors (ICIs) have revolutionized the treatment for patients with advanced-stage cancers. Since initiation of the first clinical trial of the anti-programmed cell death protein 1 (PD-1) monoclonal antibody (mAb) nivolumab in 2006, 6 mAbs targeting either PD-1 or its ligand, programmed cell death ligand 1 (PD-L1), have been approved by the US Food and Drug Administration (FDA) to treat 14 types of cancer [1]. In May 2017, the FDA granted accelerated approval to the anti-PD-1 mAb pembrolizumab for the treatment of adult and pediatric patients with unresectable or metastatic microsatellite instability-high (MSI-H) or mismatch repair-deficient (dMMR) solid tumors that continued to progress after conventional treatment, based on the data from 149 patients with MSI-H or dMMR cancers enrolled across 5 clinical trials: KEYNOTE-016, KEYNOTE-164, KEYNOTE-012, KEYNOTE-028, and KEYNOTE-158 [2]. This was the FDA’s first tissue-/site-agnostic approval, i.e., the first time the agency has approved a cancer treatment on the basis of a tumor’s specific genetic features regardless of its primary site. In December 2018, pembrolizumab was also approved for the treatment of advanced or recurrent MSI-H solid tumors that continue to progress after conventional chemotherapies in Japan. This new indication marks a paradigm shift in the therapeutic strategy of cancers; however, when considering the best indication for ICIs and their safe and effective usage, it is essential for clinicians to understand their molecular biologic background. This review highlights the nature of the DNA mismatch repair (MMR) pathway and microsatellite instability (MSI), and their implications for predicting the response to immune checkpoint blockade. In addition, we summarize the MSI status and immune checkpoint therapies in the field of gastrointestinal, hepatobiliary, and pancreatic cancers.

## DNA repair pathway and mismatch repair

Cancer is a genomic disease, and the accumulation of genetic aberrations in tumor-related genes is a critical step in malignant transformation [3]. In fact, recent innovations in next-generation sequencing (NGS) technologies have unveiled that the accumulation of genetic alterations, including nucleotide alterations and structural variations, as well as epigenetic changes such as DNA methylations and histone modifications leads to the transformation of normal cells into cancer cells [4–6]. DNA is continually exposed to endogenous and exogenous sources of damage; therefore, accurate and timely repair of DNA damage is essential for maintaining DNA fidelity and stability. Multiple pathways cooperatively function to repair different types of DNA damage. Key DNA repair pathways include base excision repair, nucleotide excision repair, MMR, homologous recombination repair, non-homologous end-joining, and interstrand crosslink repair [7]. In addition to these high-fidelity repair pathways, alternative error-prone repair pathways are available to compensate for their deficiencies [8].

**Fig. 1:**
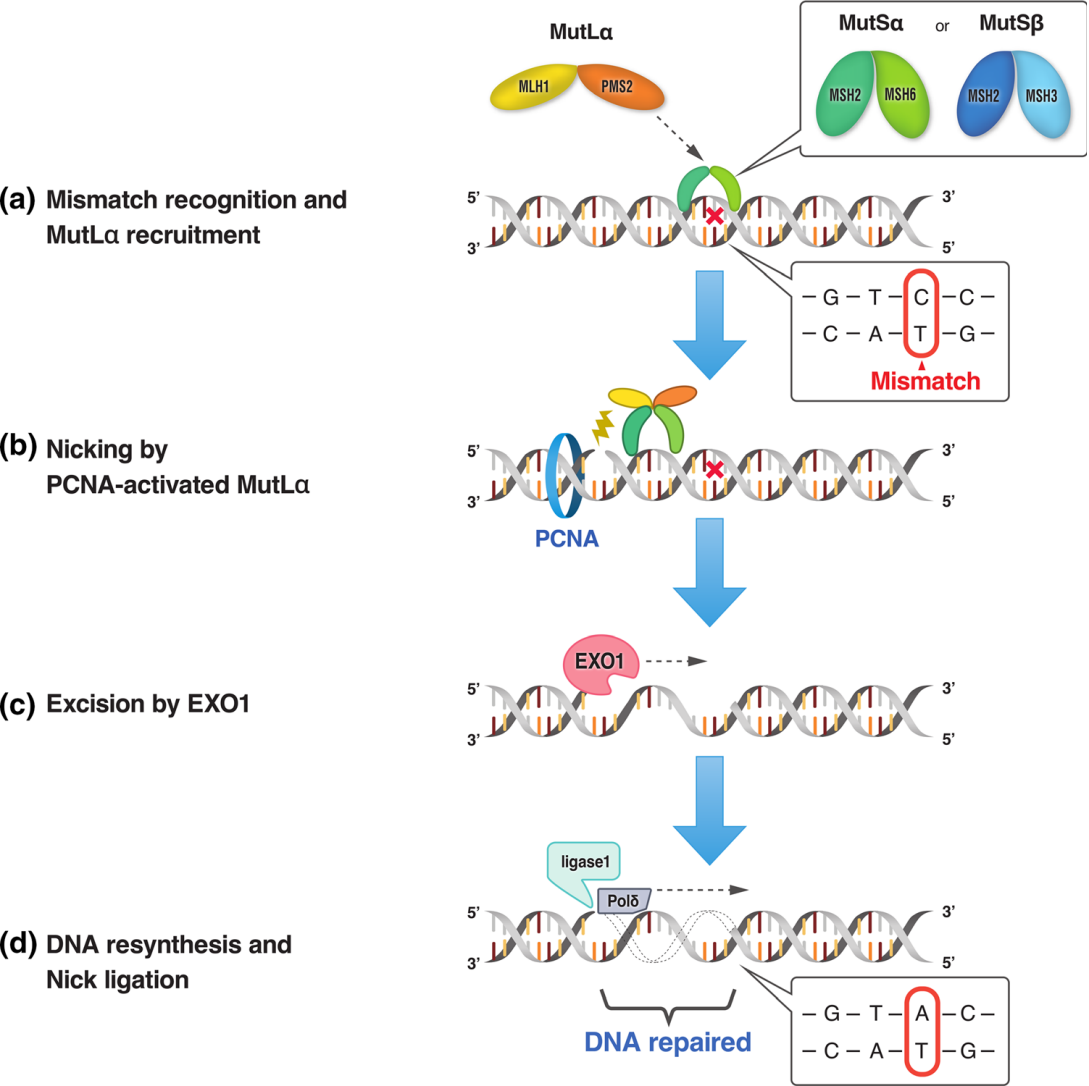
Schematic diagram of the DNA mismatch repair (MMR) pathway. The MMR pathway involves four steps: mismatch recognition, nicking, excision, and DNA resynthesis/nick ligation. **a** MutSα (heterodimer of MSH2 and MSH6 proteins) or MutSβ (heterodimer of MSH2 and MSH3 proteins) recognizes and binds to mismatches that occur during DNA replication, and subsequently recruits MutLα (heterodimer of MLH1 and PMS2 proteins). **b** Proliferating cell nuclear antigen (PCNA) activates MutLα, which makes a DNA nick 5′ to the mismatch. **c** Exonuclease 1 (EXO1) catalyzes the excision of the nascent DNA strand up to and slightly beyond the mismatch. **d** The DNA excision gap is re-synthesized by polymerase δ (Polδ) and the remaining nick is sealed by DNA ligase I

Among various DNA repair pathways, the MMR pathway plays a pivotal role in maintaining DNA replication fidelity and genome stability [9, 10]. MMR maintains genomic integrity by correcting DNA base substitution mismatch, frameshift (insertion/deletion), and slippage, conditions that are generated by DNA replication errors. In eukaryotes, MMR recognizes mismatches by two protein complexes, MutSα (heterodimer of mutS homologue 2 [MSH2] and mutS homologue 6 [MSH6] proteins) and MutSβ (heterodimer of MSH2 and mutS homologue 3 [MSH3] proteins) (Fig. 1). MutSα recognizes base substitution mismatches and small (up to 3 nucleotides) insertion/deletion loops, while MutSβ recognizes larger insertion/deletion loops up to 13 nucleotides in size and does not repair base substitutions [11]. MutSα or MutSβ binds to the mismatch in an adenosine triphosphate-dependent manner and subsequently recruits MutLα (heterodimer of mutL homologue 1 [MLH1] and postmeiotic segregation increased 2 [PMS2] proteins). MutLα forms a ternary complex with MutS at the mismatch. Proliferating cell nuclear antigen activates the latent endonuclease in the PMS2 subunit of MutLα, which makes a DNA nick at 5′ to the mismatch. After the DNA incision step, exonuclease 1 is recruited and activated by MSH2 and/or MLH1 [9]. Activated exonuclease 1 catalyzes the excision of the nascent DNA strand up to and slightly beyond the mismatch. The DNA excision gap is re-synthesized by polymerase δ stimulated by proliferating cell nuclear antigen, and the remaining nick is sealed by DNA ligase I. As described above, the MMR pathway plays an important role in maintaining DNA fidelity by repairing DNA replication errors; therefore, MMR deficiencies result in additive mutations throughout the genome and a strong hypermutator phenotype known as MSI [10].

## Microsatellite instability and cancer predisposition

**Fig. 2:**
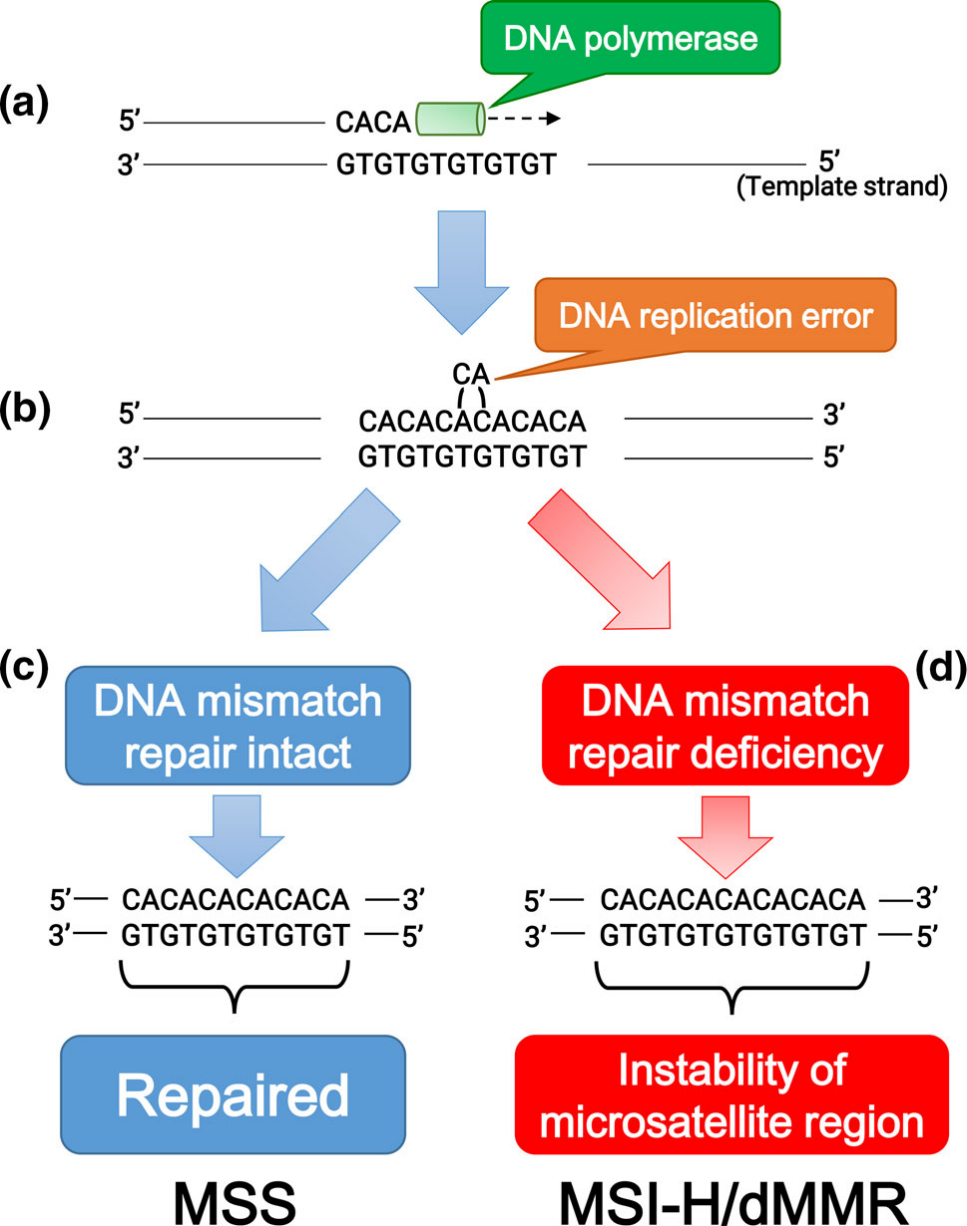
Schematic diagram of microsatellite stability (MSS) and microsatellite instability-high or mismatch repair deficiency (MSI-H/dMMR). **a** DNA polymerase initiates replication at microsatellite sequences (cytosine/adenine [CA] × 6 repeats). **b** The CA repeat is wrongly incorporated into the chain of replicated DNA due to DNA polymerase slippage during replication. **c** When DNA mismatch repair is intact, the replication error is repaired and MSS is maintained. **d** In mismatch repair deficiency, failure of elimination of the incorrectly incorporated CA repeat leads to the instability of microsatellite lesions (CA × 7 repeats)

Among human DNA sequences, there are more than 100,000 areas of short tandem repeat sequences termed microsatellites that are particularly susceptible to acquiring errors when the MMR pathway is impaired. Cells with an abnormally functioning MMR pathway are unable to correct errors during DNA replication, which causes the creation of an inconsistent number of microsatellite nucleotide repeats, leading to the instability of microsatellite regions (Fig. 2) [10]. MSI reflects the condition of genetic hypermutability that results from impaired DNA MMR, and is accompanied by a 100- to 1000-fold increase in the mutation rate [10, 12]. The presence of MSI is a sign of either sporadic or hereditary dysfunction of the MMR pathway caused by various factors, including mutations in MMR-related genes, inactivation of MMR gene transcription due to hypermethylation of its promoter region, or inflammation-mediated transcriptional repression [12–14].

Lynch syndrome (LS) is an autosomal dominant disorder that arises from germline mutations in MMR-related genes [13]. In a recent large-scale analysis, LS was identified in 16.3% (53/326), 1.9% (13/699), and 0.3% (37/14,020) of patients with MSI-H, MSI-indeterminate, and microsatellite-stable (MSS) tumors, respectively [15]. LS predisposes to various types of cancers, most frequently colorectal cancer (CRC) and endometrial adenocarcinoma [12]. LS is the most common cause of inherited CRC and accounts for approximately 2–4% of newly diagnosed CRC. Accurate estimates of the cancer risk in LS are provided by the Prospective Lynch Syndrome Database, in both individuals who have yet to develop cancer and those who have survived cancer [16]. LS is caused by heterozygous germline mutations in one of the four key MMR genes, *MLH1*, *MSH2*, *MSH6*, and *PMS2*. Although more than 1500 variants of mutations have been identified in LS, mutations in *MLH1* (40–50%) or *MSH2* (34–39%) are the main cause, while those in *MSH6* (7–18%) and *PMS2* (8%) are rare [10, 17]. Inherited deletions at the 3′-end of the *EPCAM* gene, which is located upstream of the *MSH2* allele, have been identified as another mechanism causing LS by epigenetic inactivation of the *MSH2* gene [18]. The phenotype of LS differs according to which of the MMR-related genes contains the causative mutation [13, 17]. For example, extracolonic cancers are frequently observed in cases with heterozygous *MSH2* mutation, whereas in cases with heterozygous *MLH1* mutation, CRC is dominantly observed and extracolonic cancers are less frequent than in those with *MSH2* mutations. The high incidence of various cancers in patients with LS indicates that the accumulation of mutations caused by MMR dysfunction increases the carcinogenetic risk.

## Diagnostics of microsatellite instability

Accumulating evidence suggests that stratifying patients with MSI-H/dMMR tumors or LS can facilitate personalized cancer therapy or surveillance. Indeed, several studies have demonstrated that MSI-H/dMMR is a positive predictor for response to ICIs [19]. Hence, diagnostic procedures for MSI status with high versatility and reliability are essential for the application of ICIs for cancer treatment.

Two standard procedures are used to diagnose MSI status, immunohistochemistry (IHC) and polymerase chain reaction (PCR)-based testing. In addition, the utility of NGS-based analysis was recently reported [20]. IHC is a widely available and less expensive method for MSI analysis than other tests. Another advantage of IHC is that testing four representative MMR-related proteins (MLH1, MSH2, MSH6, and PMS2) can direct germline testing to that specific gene and assist in the identification of patients with LS [21]. IHC is reported to be highly concordant with PCR-based testing with a sensitivity of > 90% and nearly perfect specificity [22]. IHC lacks a little sensitivity for identifying MSI, however, because in some cases with missense mutations in MMR-related genes, the corresponding MMR protein expression remains intact but is functionally inactivated [23].

Genotyping of microsatellites by PCR-based testing is another standard method for diagnosing MSI status. DNA mismatches caused by MMR dysfunction commonly occur in microsatellite regions. Therefore, MMR deficiency through qualitative or quantitative protein abnormalities results in the expansion or contraction of microsatellite regions, which can be utilized as “microsatellite markers” for PCR-based MSI testing [10]. The Bethesda Guidelines recommended the National Cancer Institute (NCI)-approved panel of five microsatellite markers (the “Bethesda panel”) for MSI testing, including two mononucleotide repeats (BAT-25 and BAT-26) and three dinucleotide repeats (D2S123, D5S346, and D17S250) [24]. These regions are amplified from both tumor and normal DNA by multiplex PCR, and their sizes are assessed by capillary electrophoresis [25]. If two or more of the five markers are shifted in comparison with normal DNA, the tumor is defined as the MSI-H phenotype. In a follow-up NCI workshop, however, several limitations of the Bethesda panel were identified due to the inclusion of the three dinucleotide markers [26]. Employing a panel of five quasi-monomorphic mononucleotide-repeat markers in a pentaplex PCR obviates the need for obtaining normal control DNA, and exhibits better sensitivity in comparison with the Bethesda panel [27, 28]. Wong et al*.* compared the sensitivity and specificity in a series of 80 endometrial tumors and revealed that the Bethesda panel and the pentaplex PCR panel of five mononucleotide-repeat markers detected the same subset of 21 MSI-H tumors [28]. They found, however, that the pentaplex panel detected the instability in 101 out of 105 (96%) markers as compared with the instability in 84 out of 105 (77%) markers detected by the Bethesda panel in MSI-H tumors. The Japan Pharmaceuticals and Medical Devices Agency approved a companion diagnostic for MSI-H using five quasi-monomorphic mononucleotide-repeat markers (FALCO Biosystems Ltd., Kyoto, Japan) at the same time as approval of pembrolizumab for the treatment of MSI-H solid tumors.

The NGS-based pan-cancer approach is an alternative method for MSI determination [20]. Several studies with different NGS platforms demonstrated that the NGS-based method is 95.8–100% concordant with PCR-based testing [29, 30]. The NGS-based approach has several advantages over other methods. First, it can detect genomic alterations and copy number alterations in a large number of cancer-related genes, which can lead to identifying candidate molecular targeted therapies. Second, it also shows the tumor mutation burden (TMB, the total number of mutations per coding area of a tumor genome). Third, the NGS-based approach can decrease the demand for tumor tissue samples.

## Clinical importance of MSI and/or TMB in gastrointestinal malignancies

**Fig. 3:**
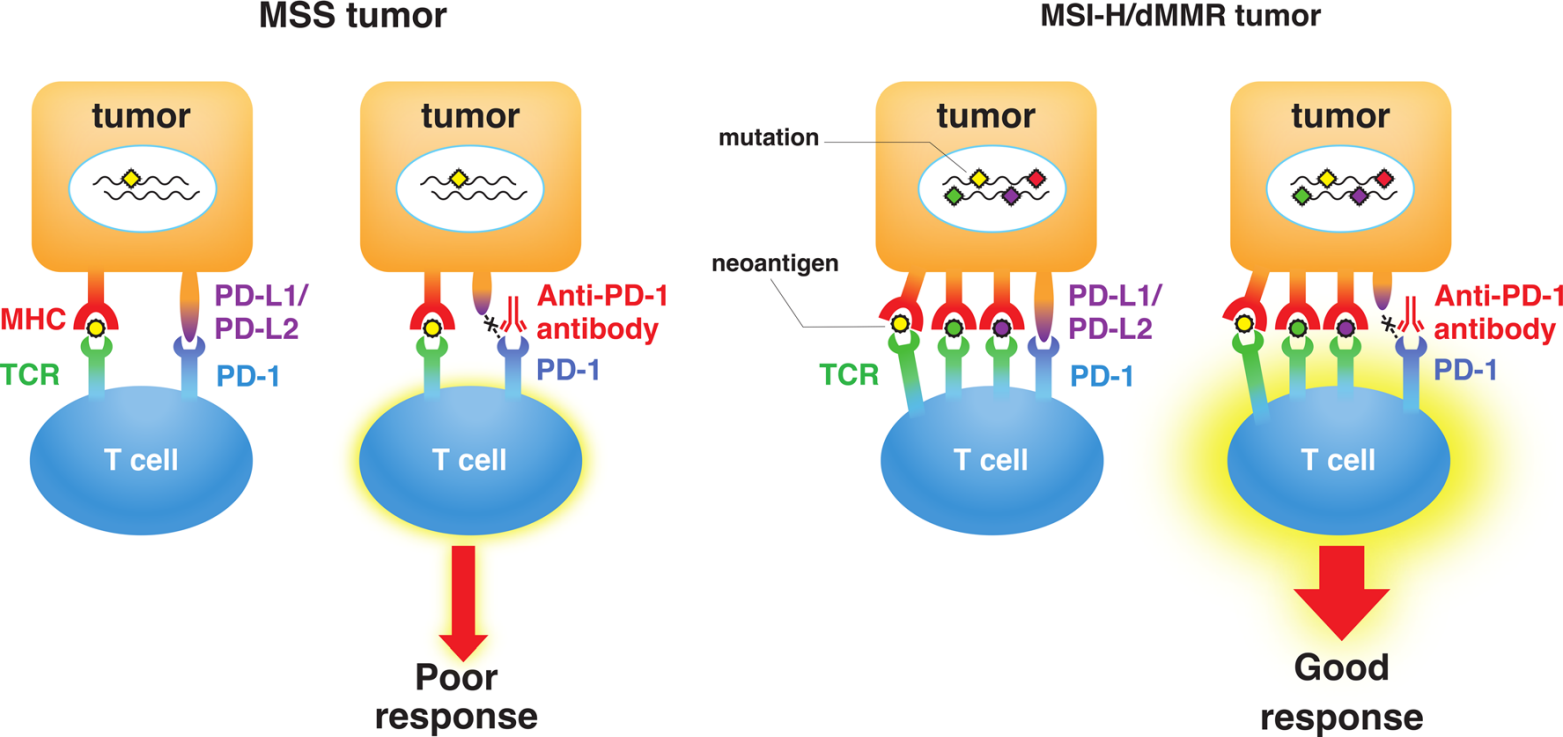
Difference in the response to immune checkpoint therapy between microsatellite-stable (MSS) tumors and microsatellite instability-high or mismatch repair deficiency (MSI-H/dMMR) tumors. High mutation burden (rhombuses) in MSI-H/dMMR tumor leads to the synthesis of mutation-associated neoantigens (small circles) presented by major histocompatibility complex (MHC) class I molecules, which attracts cytotoxic T lymphocytes to the tumor microenvironment via T cell receptor (TCR) engagement with MHC. Blockade of the programmed cell death protein 1 (PD-1)–programmed cell death ligand 1 (PD-L1) interaction with an anti-PD-1 antibody results in T cell activation and infiltration into the tumor, leading to an objective tumor response

Llosa and colleagues first reported that CRCs with a high infiltration of activated CD8+ cytotoxic T lymphocytes (CTLs) as well as activated Th1 cells characterized by interferon-γ production had dMMR [31]. They also observed that upregulation of immune checkpoint proteins including PD-1 and PD-L1 in advanced MSI-H/dMMR tumors, which explains why MSI-H/dMMR tumors are not naturally eliminated despite hostile CTL/Th1 microenvironments. Most significantly, their report suggested the utility of MSI status as a predictive marker for the response to PD-1/PD-L1 blockade in cancer patients (Fig. 3). Follow-up studies revealed a correlation between MSI status, TMB, and clinical response to treatments with ICIs in various cancers [32–34]. High TMB leads to the synthesis of aberrant and potentially immunogenic mutation-associated neoantigens by the cancer cells, which attract CD8+ CTLs and activated Th1 cells to the tumor microenvironment [32]. Furthermore, there is a significant correlation between TMB and the response to anti-PD-1/PD-L1 therapy across various types of cancer [19, 33, 34]. Diaz et al*.* reported the results of the phase 2 KEYNOTE-158 basket study, in which 77 patients with MSI-H non-CRC across 20 tumor types (52% with ≥ 1 prior therapies) were enrolled, including endometrial (*n* = 17), gastric (*n* = 11), small intestinal (*n* = 10), pancreatic (*n* = 9), and biliary tract (*n* = 8) cancers [35]. The objective response rate (ORR) was 37.7% [95% confidence interval (CI) 26.9–49.4], and the 6-month overall survival (OS) and progression-free survival (PFS) rates were 73% and 45%, respectively. Furthermore, Samstein et al*.* recently reported an analysis of the clinical and NGS-based genomic data of 1662 patients with advanced cancer, and demonstrated that high TMB is associated with improved survival in patients receiving treatments with ICIs across a wide variety of cancer types [36]. Interestingly, Shen et al*.* recently reported that deficiency of AT-rich interaction domain 1A (ARID1A), a subunit of the chromatin remodeling complex SWI/SNF, led to impaired MMR and treatment with ICIs resulted in the prolonged survival of mice bearing ARID1A-deficient tumors [37].

As the clinical importance of MSI status and TMB has become broadly recognized, efforts have been made to understand the landscape of MSI status and TMB across various cancer types by NGS-based methods (Table 1). Recent whole-exome sequencing data analyses revealed an MSI landscape among various cancer types [38–41]. Regarding gastrointestinal malignancies, 16.6–19% of colon cancers and 7.5–21.9% of gastric cancers (GCs) were identified as MSI-H. Rectal cancers had a lower prevalence of MSI-H (2.2–9.2%). On the other hand, the rate of MSI-H in hepatocellular carcinomas (HCCs) was less than 3%; however, some population of microsatellites was unstable with a high frequency in HCCs classified as MSS. Nakamura et al*.* recently reported the TMB in tissue samples from 1759 advanced gastrointestinal, hepatobiliary, and pancreatic tumors using the Oncomine Cancer Research Panel as a part of the Nationwide Cancer Genome Screening Project in Japan [42]. High TMB was defined as more than 20 mutations per megabase. In CRC (*n* = 751), high TMB was observed in 23.6%, including 75.0% of MSI-H and 17.1% of non-MSI-H tumors. In non-CRC, high TMB was observed in 13.3% of GC, 17.5% of esophageal cancer, 27.9% of pancreatic cancer, 26.1% of biliary tract cancer (BTC), 30.0% of small intestinal cancer, 6.9% of gastrointestinal stromal cancer, 7.4% of HCC, and 14.8% of neuroendocrine tumor/cancer [42]. TMB analysis may be useful as an agnostic histologic indicator to identify patients who can benefit from ICIs; however, a universal definition of high TMB may be difficult to establish because the TMB cut-points associated with improved survival varies between cancer types [36].

**Table 1:** Frequencies of microsatellite instability-high and/or high tumor mutation burden among gastrointestinal, pancreatic, and hepatobiliary cancers

Tumor type	dMMR/MSI-H (%)	High TMB (%)	References
Esophageal cancer	0–3.3	3.5–17.5	[38–41, 45]
Gastroesophageal junction cancer	4–8	3.1	[40, 43]
Gastric cancer	7.5–21.9	8.3–13.3	[38–43]
Small intestinal cancer	12	10.2–30.0	[40, 42]
Gastrointestinal stromal cancer	0	0–6.9	[40, 42]
Right-sided colon cancer	13.5–27	14.6	[38–43]
Left-sided colon cancer	2.0–2.2	3.5	[40, 43]
Rectal cancer	2.2–9.2	3.0	[38–41, 43]
Pancreatic cancer	0–1.3	1.4–27.9	[39–41, 43, 60, 61]
Biliary tract cancers	0–3	3.7–26.1	[10, 40–42, 66]
Hepatocellular carcinoma	0–2.9	2.2–7.4	[38–42, 70]
Neuroendocrine tumor/cancer	0	1.3–14.8	[40, 42]

*dMMR* mismatch repair deficient, *MSI-H* microsatellite instability-high, *TMB* tumor mutation burden

From the next section, we discuss the MSI status and treatment with ICIs for esophageal, gastrointestinal, pancreatic, and hepatobiliary cancers. The results of representative clinical studies showing the efficacy of ICIs in association with MSI status in gastrointestinal cancers are summarized in Table 2.

**Table 2:** Representative clinical studies showing the efficacy of immune checkpoint inhibitors in association with MSI status in gastrointestinal cancers

Tumor type	Treatment	Phase	Trial	Patient feature	Clinical outcome	References
Gastric and Gastroesophageal junction cancer	Pembrolizumab	2	NCT02589496	61 patients with metastatic GC 7 patients with MSI-H 54 patients with MSS	ORR: 24.6% (total) DCR: 57.4% ORR of MSI-H: 85.7% (6/7) ORR of MSS: 16.7% (9/54)	[48]
	Pembrolizumab	2	KEYNOTE-059 (NCT02335411)	259 patients with advanced GC/GEJc with ≥ 2 prior lines of treatment 7 patients with MSI-H 167 patients with MSS	ORR: 11.6% (total) ORR of MSI-H: 57.1% (4/7) ORR of MSS: 9.0% (15/167)	Fuchs et al JAMA Oncol 2018;4:e180013
Colorectal cancer	Pembrolizumab	2	KEYNOTE-016 (NCT01876511)	28 patients with dMMR CRC 25 patients with pMMR CRC	dMMR vs pMMR ORR: 50% vs 0% DCR: 89% vs 16%	Le et al ASCO #103, 2016
	Pembrolizumab	2	KEYNOTE-164 (NCT02460198)	63 patients with MSI-H/dMMR mCRC with ≥ 1 prior line of therapy	ORR: 58% (2 CRs and 18 PRs)	[53]
	Nivolumab + low-dose Ipilimumab	2	CheckMate-142 (NCT02060188)	Preciously treated 119 patients with MSI-H/dMMR mCRC	ORR: 58% DCR: 81%	[54]

*MSI*, microsatellite instability, *GC* gastric cancer, *MSI-H* microsatellite instability-high, *MSS* microsatellite stable, *ORR* objective response rate, *DCR* disease control rate, *GEJc* gastroesophageal junction cancer, *dMMR* mismatch repair deficient, *CRC* colorectal cancer, *pMMR* mismatch repair proficient, *ASCO* American Society of Clinical Oncology, *mCRC* metastatic colorectal cancer, *CR* complete response, *PR* partial response

## MSI status and treatment with ICIs for gastric cancer

Among all cancer types, gastrointestinal adenocarcinomas exhibit MSI properties at a comparatively high proportion. Comprehensive molecular analysis of gastrointestinal adenocarcinomas revealed that MSI-H adenocarcinomas are observed primarily in the distal stomach and proximal colon [43]. The Cancer Genome Atlas Research Network analyses demonstrated that gastric and gastroesophageal junction adenocarcinomas are divided into four subtypes according to their molecular features: tumors exhibiting chromosomal instability (CIN), MSI-H, Epstein–Barr virus (EBV) positive, and genomically stable [44, 45]. Among them, MSI-H tumors account for approximately 22% of GCs, and a small minority of MSI-H GCs are related to a germline mutation in MMR-related genes [43]. Pathophysiologically, MSI-H GCs are linked with female sex, older age, intestinal type, and distal location, and almost all sporadic MSI-H GCs exhibit epigenetic silencing of *MLH1* in the context of a CpG island methylator phenotype [43, 46]. Interestingly, the MSI phenotype is established in cancer cells at the early stage of non-hereditary, sporadic GC development [47]. MSI-H GCs have a high incidence of somatic mutations, including mutations in genes related to receptor tyrosine kinase-RAS signaling, but generally lack targetable alterations compared with CIN-type GCs having therapeutic targetable amplification in receptor tyrosine kinase. Importantly, MSI-H- or EBV-positive GCs have a high interferon-γ gene expression signature levels and are highly correlated with PD-L1 positivity [43, 48]. Therefore, advanced MSI-H GCs with metastases could be suitable targets of anti-PD-1 therapies. Strikingly, Kim et al*.* reported that patients with MSI-H- and EBV-positive metastatic gastric cancer had dramatic responses to pembrolizumab [48]. ORR was 85.7% in patients with MSI-H tumor and 100% in those with EBV-positive tumor, compared with 6.3% in those with other types of tumor. These results imply the importance of MSI and EBV testing in the choice of therapy for gastric cancer.

## MSI status and treatment with ICIs for colorectal cancer

CRCs are divided into hypermutated types and non-hypermutated types by The Cancer Genome Atlas analyses [49, 50]. Among hypermutated types that account for 16% of colorectal cancers, one-quarter of those with mutations in the proofreading (exonuclease) subunit of polymerase epsilon have an extremely high incidence of somatic mutations, and three-quarters exhibit MSI-H, usually with *MLH1* promoter methylation and a CpG island methylator phenotype. The Colorectal Cancer Subtyping Consortium classified CRCs into four consensus molecular subtypes (CMSs) with distinguishing biologic features: CMS1 (MSI immune, 14%), CMS2 (canonical, 37%), CMS3 (metabolic, 13%), CMS4 (mesenchymal, 23%), and mixed features (13%) [51]. Among them, CMS1 tumors exhibit MSI-H features with *MLH1* promoter methylation or mutations in MMR-related genes. The large proportion of CRCs arises from adenoma with inactivated mutation or deletion in the tumor suppressor gene *APC* (adenoma-carcinoma sequence); however, MSI-H tumors develop via a different pathway. Inherited MSI-H CRCs occur due to germline mutations in MMR-related genes such as *MLH1* and *MSH2*, whereas sporadic MSI-H CRCs typically arise from sessile-serrated adenomas/polyps with *BRAF* V600E mutation and widespread hypermethylation, including *MLH1* promoter methylation (serrated pathway). MSI-H CRCs are frequently diagnosed in the right-side colon and have similar pathologic features, regardless of inherited and sporadic tumor types. These cancers have increased tumor-infiltrating lymphocytes, mainly comprising Th1 and CTLs, and high expression of PD-L1, along with strong activation of immune evasion pathways [51, 52]. Although recurrent MSI-H CRCs have a worse prognosis, these tumors are a potential target for anti-PD-1 therapy.

Le et al*.* recently reported the data from cohort B of the phase 2 KEYNOTE-164 study investigating the antitumor activity of pembrolizumab for patients with metastatic MSI-H CRC treated with ≥ 1 prior line of therapy [53]. Of 63 patients enrolled, the ORR was 32% (95% CI 21–45) with 2 complete responses and 18 partial responses. The 12-month PFS rate was 41% and the 12-month OS rate was 76%. In addition, the result of a long-term follow-up (median 25.4 months) of patients with previously treated metastatic MSI-H/dMMR CRC enrolled in the phase 2 CheckMate-142 trial, nivolumab plus low-dose ipilimumab (anti-CTL-associated antigen 4 [CTLA-4] mAb, 1 mg/kg), was recently presented at ASCO GI 2019 [54]. The ORR and disease control rates were 58% (69 of 119 patients, 95% CI 49–67) and 81% (96 of 119 patients, 95% CI 72–87), respectively.

## MSI status and treatment with ICIs for pancreatic cancer

Pancreatic ductal adenocarcinoma (PDAC) is a lethal cancer with an extremely poor prognosis [55]. Unfortunately, ICIs including anti-PD-1 or anti-CTLA-4 mAb alone or in combination exhibit little efficacy against PDAC [56–58]. Its poor response to immune therapies results from an immunosuppressive microenvironment, poor T cell infiltration, and a low TMB. The MSI-H/dMMR phenotype is indeed very rare in PDAC [10, 59]. Hu et al*.* reported that the dMMR phenotype was present in only 0.8% (7/833) of patients with PDAC [60]. Lupinacci et al*.* also reported a retrospective and multicenter study of MSI status in 443 cases with PDAC including 58 intraductal papillary mucinous neoplasm (IPMN)-associated PDACs [61]. In their report, the MSI-H/dMMR phenotype was present in 5 of 385 (1.3%) non-IPMN-associated PDACs and 4 of 58 (6.9%) IPMN-associated PDACs. PDAC has minimal-to-moderate infiltration of CD3, CD4, and CD8 T cells; however, the infiltrates are predominantly present in the stromal area of the tumor and are excluded from the tumoral area of PDACs [62]. Furthermore, metastatic PDACs had lower T cell infiltration compared with resectable primary PDACs; therefore, advanced PDACs have poor immunogenicity. To increase the responsivity of PDAC to ICIs, it is necessary to elucidate the mechanisms of increasing initial T cell priming, overcoming the immunosuppressive tumor microenvironment, and inhibiting compensatory mechanisms of T cell anergy and exhaustion [58]. Blando et al*.* recently reported the presence of a high number of CD68+ macrophages in the tumor stromal area [62]. Moreover, V-domain immunoglobulin suppressor of T cell activation (VISTA) was predominantly expressed on the macrophages. An activated VISTA pathway decreases T cell responses in the tumor to a greater degree than PD-L1 inhibition, suggesting that PD-1/PD-L1 inhibition might fail in the treatment of PDACs because an untreated VISTA pathway still suppresses the immune response. Combination therapy to increase T cell infiltration, possibly using anti-CTLA-4 mAb plus anti-VISTA antibody to target macrophages, may be a prominent treatment strategy for PDAC. Although the MSI-H/dMMR phenotype is very rare in PDAC, the American Society of Clinical Oncology clinical practice guideline recommends routine testing for MSI-H or dMMR, and treatment with pembrolizumab as second-line therapy for patients testing positive for MSI-H or dMMR [63]. The National Comprehensive Cancer Network guidelines Version 1.2019 also recommends MSI and/or MMR testing in patients with locally advanced or metastatic PDAC, and treatment with pembrolizumab only for MSI-H or dMMR tumors [64].

## MSI status and treatment with ICIs for biliary tract cancer

BTCs are often diagnosed at an advanced stage, and the standard chemotherapy regimen gemcitabine plus cisplatin provides limited benefit [65]. Therefore, it is important to investigate the treatment response of ICIs against BTCs and identify a predictive response marker. The rate of MSI-H/dMMR BTCs is reported to be 1–3% [10]. Although MSI-H BTCs are rare, anti-PD-1/PD-L1 mAbs exert a certain antitumor activity in a subset of advanced BTCs. Ueno et al*.* reported the results of the phase 2, multicohort KEYNOTE-158 study evaluating the antitumor activity and safety of pembrolizumab in 104 patients with advanced BTC and prior progression/intolerance on standard therapy [66]. Among 99 patients in whom MSI status was evaluated, none were MSI-H. An evaluation of PD-L1 expression by IHC assay revealed that 61 of 95 tumor samples expressed PD-L1 expression. The ORR was 6.6% (95% CI 1.8–15.9) and 2.9% (0.1–15.3) among patients who were PD-L1 positive and negative, respectively. The median PFS was 1.9 months (1.8–2.0) vs 2.1 months (1.9–2.6), and the median OS was 7.2 months (5.3–11.0) and 9.6 months (5.4–12.8) among patients who were PD-L1 positive vs PD-L1 negative, respectively. Two patients with PD-L1-positive tumors showed a long-term response period of more than 15 months. Although the OS and PFS of pembrolizumab as a second-line therapy are not fully satisfactory, it is worth considering because no standard salvage chemotherapy regimen for advanced BTCs in progression after gemcitabine and platinum compounds has yet been identified. The results of the phase 1 study (JapicCTI-153098) investigating the safety and tolerability of nivolumab monotherapy or in combination with cisplatin plus gemcitabine for patients with unresectable or recurrent BTC suggested that nivolimab had a manageable safety profile and signs of clinical activity [67]. Additionally, a recent report of the phase 1 study of durvalumab (anti-PD-L1 mAb) with or without tremelimumab (anti-CTLA-4 mAb) suggested that their combination might become a promising regimen for patients with advanced BTC after conventional chemotherapy [68].

## MSI status and treatment with ICIs for hepatocellular carcinoma

After a decade with sorafenib as the only available multi-targeted tyrosine kinase inhibitor (TKI) for HCC, regorafenib as second-line therapy and lenvatinib as another first-line therapeutic agent were finally approved [69]. The prognosis of HCC is still poor, however, because of the high potential for intra- and extra-hepatic multiple recurrence and metastasis. Goumard et al. analyzed 122 patients with HCC and found no tumors displaying a typical MSI-H phenotype as defined by PCR-based MSI testing [70]. Low levels of MSI, however, were observed in 31.1% (38/122) of HCCs. Furthermore, the rate of MSI was higher in patients with cirrhosis than in those without cirrhosis [70]. Some degree of MSI is known to be induced by chronic inflammation, as reported in pancreatitis [71] and ulcerative colitis [72]. We previously demonstrated that proinflammatory cytokine stimulation induced transcriptional downregulation of *MSH2* via inflammation-mediated microRNA-21 expression in hepatocytes [14]. Furthermore, hepatocyte-specific disruption of *MSH2* in mice results in the development of liver tumors with the histologic features of HCC. Therefore, although the MSI-H phenotype is rare in HCC, inflammation-mediated dysfunction of the MMR pathway can contribute to an accumulation of mutations during hepatitis-associated tumorigenesis. In fact, the CheckMate-040 study revealed that nivolumab induced durable responses in both sorafenib-naïve patients (ORR: 23%, disease control rate: 63%) and sorafenib-experienced patients (ORR: 16–19%) with advanced HCC [73]. In September 2017, nivolumab was approved by the FDA as a second-line treatment for HCC after sorafenib failure based on a 154-patient subgroup analysis of CheckMate-040 [74]. However, a randomized phase 3 study evaluating nivolumab versus sorafenib as a first-line treatment in patients with unresectable HCC (CheckMate-459) recently revealed that the trial did not achieve statistical significance for its primary endpoint of OS per the pre-specified analysis [hazard radio (HR) 0.85 (95% CI 0.72–1.02); *p* = 0.0752] [75]. Pembrolizumab was also granted accelerated approval by the FDA in November 2018, as a second-line treatment after sorafenib failure based on the data from the phase 2 KEYNOTE-224 trial [76]. The results from the phase 3 KEYNOTE-240 trial, however, demonstrated that although patients treated with pembrolizumab as a second-line treatment achieved a longer OS (HR = 0.78; 95% CI 0.611–0.998; *p* = 0.0238) and PFS (HR = 0.78; 95% CI 0.61–0.99; *p* = 0.0219) versus placebo, the findings were not deemed statistically significant as per the prespecified statistical plan [77]. Therefore, ICI treatment in combination with TKI or different types of ICI may be promising in the future, rather than the strategy of sequential therapy from TKI to ICI [78]. There are various ongoing trials investigating anti-PD-1/PD-L1 mAb in combination with lenvatinib, bevacizumab, or anti-CTLA-4 mAb tremelimumab [79].

## Future prospects for MSI testing and precision cancer medicine

An NGS-based comprehensive approach is undergoing a paradigm shift in cancer diagnosis and treatment strategy construction. One of the NGS-based comprehensive genomic profiling assays, FoundationOne CDx™ (Foundation Medicine Inc. and Chugai Pharmaceutical Co. Ltd.) is the first FDA-approved broad companion diagnostic that is clinically and analytically validated for solid tumors. The Pharmaceutical Affairs and Food Sanitation Council of the Ministry of Health, Labour and Welfare of Japan also approved FoundationOne CDx™ and OncoGuide™ NCC Oncopanel System (Sysmex, Kobe, Japan) in December 2018. FoundationOne CDx™ can detect not only genomic alterations in 324 genes known to drive cancer growth, but also MSI status and TMB using DNA isolated from formalin-fixed paraffin-embedded tumor tissues [80]. As mentioned earlier, an NGS-based comprehensive approach can decrease the demand for tumor tissue samples, as well as shorten the period from test to treatment. In some cases, however, obtaining tumor tissue samples may be difficult due to poor patient condition or a tumor that is difficult to access. Furthermore, recent studies of multi-region NGS analysis revealed intra- and inter- tumor genomic heterogeneities in various types of cancers [47, 81–84]. The needle biopsy method examines only a tiny fraction of a tumor, potentially influencing the interpretation of NGS assay results. Liquid biopsy may overcome these limitations of needle biopsy-based analysis. Liquid biopsy is a minimally invasive procedure compared with tumor biopsy that analyzes circulating tumor DNA (ctDNA). ctDNA is a fragmented DNA released from cancer cells into the blood. Recent progress in amplicon-based NGS assays has increased the sensitivity and specificity for the detection of the mutant alleles in the liquid biopsy method, which supports the potential of liquid biopsy for diagnosis, early detection, selection of therapy, and monitoring the response to therapy [85]. One liquid biopsy-based NGS assay, Guardant360^®^ (Guardant Health, CA, USA), can identify clinically relevant genomic alterations (base substitutions, insertions and deletions, amplifications, and fusions) in 73 commonly altered oncogenes, as well as MSI status [86]. Further studies are needed to overcome the current challenges of sample preparation, standardization of techniques, reliable data interpretation, and acceptance in clinical practice. The combination of medicinal innovation and NGS-based assays as well as the construction of a biobank of high quality would lead to a paradigm shift in diagnosis and treatment for cancer, and mark a new era of precision cancer medicine using ICIs.

## Conclusions

The approval of anti-PD-1 mAb for the treatment of MSI-H/dMMR tumors marked the first step toward revolutionizing cancer treatment strategies. MSI status is currently considered a practical surrogate marker for immunotherapeutic response; however, further studies are needed to investigate more precise biomarkers such as TMB or detection of immunogenic neoantigens, which will significantly advance precision cancer medicine.

## Abbreviations

ARID1A: AT-rich interaction domain 1A
BTC: Biliary tract cancer
CI: Confidence interval
CIN: Chromosomal instability
CMS: Consensus molecular subtype
CRC: Colorectal cancer
ctDNA: Circulating tumor DNA
CTL: Cytotoxic T lymphocyte
CTLA-4: Cytotoxic T lymphocyte-associated antigen 4
dMMR: Mismatch repair deficient
EBV: Epstein–Barr virus
FDA: Food and drug administration
GC: Gastric cancer
HCC: Hepatocellular carcinoma
HR: Hazard ratio
ICI: Immune checkpoint inhibitor
IHC: Immunohistochemistry
IPMN: Intraductal papillary mucinous neoplasm
LS: Lynch syndrome
mAb: Monoclonal antibody
MLH1: MutL homologue 1
MMR: Mismatch repair
MSH2: MutS homologue 2
MSH3: MutS homologue 3
MSH6: MutS homologue 6
MSI: Microsatellite instability
MSI-H: Microsatellite instability-high
MSS: Microsatellite-stable
NCI: National Cancer Institute
NGS: Next-generation sequencing
ORR: Objective response rate
OS: Overall survival
PCR: Polymerase chain reaction
PD-1: Programmed cell death protein 1
PDAC: Pancreatic ductal adenocarcinoma
PD-L1: Programmed cell death ligand 1
PFS: Progression-free survival
PMS2: Postmeiotic segregation increased 2
TKI: Tyrosine kinase inhibitor
TMB: Tumor mutation burden
VISTA: V-domain immunoglobulin suppressor of T cell activation
